# A scenario of mitochondrial genome evolution in maize based on rearrangement events

**DOI:** 10.1186/1471-2164-11-233

**Published:** 2010-04-09

**Authors:** Aude Darracq, Jean-Stéphane Varré, Pascal Touzet

**Affiliations:** 1 Laboratoire de Genetique et Evolution des Populations Vegetales, UMR CNRS 8016, Universite Lille 1, 59655 Villeneuve d'Ascq Cedex, France; 2 Laboratoire d'Informatique Fondamentale de Lille, UMR CNRS 8022, Universite Lille 1, 59655 Villeneuve d'Ascq Cedex, France; 3 INRIA Lille-Nord Europe, 59650 Villeneuve d'Ascq, France

## Abstract

**Background:**

Despite their monophyletic origin, animal and plant mitochondrial genomes have been described as exhibiting different modes of evolution. Indeed, plant mitochondrial genomes feature a larger size, a lower mutation rate and more rearrangements than their animal counterparts. Gene order variation in animal mitochondrial genomes is often described as being due to translocation and inversion events, but tandem duplication followed by loss has also been proposed as an alternative process. In plant mitochondrial genomes, at the species level, gene shuffling and duplicate occurrence are such that no clear phylogeny has ever been identified, when considering genome structure variation.

**Results:**

In this study we analyzed the whole sequences of eight mitochondrial genomes from maize and teosintes in order to comprehend the events that led to their structural features, i.e. the order of genes, tRNAs, rRNAs, ORFs, pseudogenes and non-coding sequences shared by all mitogenomes and duplicate occurrences. We suggest a tandem duplication model similar to the one described in animals, except that some duplicates can remain. This model enabled us to develop a manual method to deal with duplicates, a recurrent problem in rearrangement analyses. The phylogenetic tree exclusively based on rearrangement and duplication events is congruent with the tree based on sequence polymorphism, validating our evolution model.

**Conclusions:**

This study suggests more similarity than usually reported between plant and animal mitochondrial genomes in their mode of evolution. Further work will consist of developing new tools in order to automatically look for signatures of tandem duplication events in other plant mitogenomes and evaluate the occurrence of this process on a larger scale.

## Background

All organelle genomes found in mitochondria of plant or animal cells are considered to have originated from an endosymbiotic form of α-Proteobacteria, and given rise to the emerging eukaryotic cell more than 10^9 ^years ago [1]. Despite their monophyletic origin, animal and plant mitochondrial genomes (mitogenomes) exhibit contrasted features, when considering size, compactness, mutation rate and gene-order variation [2]. Most animal mitogenomes are circular and compact, share the same gene content and have a size that does not exceed 20 kb. The high nucleotide mutation rate of their coding sequences has been commonly used in population genetic and phylogenetic studies [3]. However, in taxonomic studies, the introduction of gene-order variation to resolve specific nodes has proved to be a powerful tool [4]. In these animal rearranged mitogenomes, most gene rearrangements were due to inversions and translocations. But duplication events were also identified: in some cases, they were distant in the genomes, with or without loss of parts of the duplicated fragment [5]. In other cases, duplications occurred in tandem repeat and were followed either by non-random duplicate loss (cases of genes conserved side by side in the same orientation [6,7]) or by random loss (known as TDRL, Tandem Duplication with Random Loss) [8-10]. In most cases, when duplication involved a protein coding gene, only one functional copy remained.

In contrast, plant mitogenomes exhibit larger size (most are from 200 to 700 kb) and are less compact than their animal counterparts due to the occurrence of non-coding sequences and duplicated fragments. Moreover, plant mitogenomes are known to evolve rapidly in structure and slowly in sequence [11]. The occurrence of large repeated sequences has led to the idea of a complex genome, composed of alternative master chromosomes and sub-genomic molecules due to intragenomic recombination [12], even though whole sequenced genomes are usually represented as circular master circles [13,14]. At the intra-specific level, recombination through small repeat sequences is believed to be responsible for large gene-order shuffling and the emergence of new open reading frames, some of which have been involved in Cytoplasmic Male Sterility (CMS) [14,15]. In this context, the acquisition of whole sequence data for several mitogenomes found in a species opens new venues toward a better understanding of the evolutionary dynamics of this peculiar genome, especially when focusing on its high structural rearrangement rate and the origin of duplicated fragments.

The comparison of whole genomes using gene order has been an active field of research since the early 1990s. The first methods focused on the study of the minimal number of rearrangement events, mostly inversions, to go from one genome to another [16,17]. The resulting scenario could be seen as a putative evolutionary scenario. Phylogenetic reconstruction methods based on rearrangement events have also been proposed in order to compute scenarios and putative ancestors for a set of genomes [18,19]. Methods to study rearrangements that take duplicates into account have been investigated over the past decade. Since most of the mathematical models used to compute rearrangement distances and scenarios are based on the assumption that each gene or synteny block appears exactly once in each genome, methods designed for genomes without duplicates cannot be applied directly to plant mitochondrial genomes. One possible approach consists in keeping only one of the duplicates and removing the others from the genomes in order to obtain a dataset with one copy of each gene per genome [20,21]. The drawback of this solution is its high combinatorics if the number of duplicates is large. Moreover it does not provide any kind of explanation about duplication events. Other methods focus on the study of gene families, i.e. the evolutionary history of a gene and its duplicates [22]. The aim of these methods is to find the duplication events within a given phylogenetic tree. It follows that currently no method is able to reconstruct a rearrangement phylogenetic tree of genomes with duplicates. Therefore the 'manual approach' has to be used for resolving this type of evolutionary history [23].

Recently, Allen and colleagues [24] reported the whole sequencing of 5 mitogenomes in maize. As expected, the mitogenomes exhibited a large variation in size (from 535 to 740 kb) due mainly to large duplicated fragments, and gene shuffling was such that no clear evolutionary scenario could be pictured. However, on the basis of nucleotide divergence, groups of related mitogenomes could be defined and qualified as ancestral or derived though no phylogeny could be established. In the present study, we added three newly available whole mitogenome sequences of teosintes, species that are relatives of maize, to the five mitogenomes studied by Allen and colleagues [24] in order to conduct a phylogenetic analysis and ultimately comprehend the events that led to their structural features: sequence order and duplicates.

The analysis based on sequence polymorphism among the eight mitogenomes enabled us to build a robust reference tree for subsequent analyses solely based on genome structure information (sequence-order). We showed that mitogenome rearrangements could result from a mechanism similar to that found in animals, i.e. tandem duplication, but where some duplicates were partially lost. Using this evolution model, we developed a methodology to reconstruct a phylogeny based on rearrangement events that integrated most duplicates, and ended up with an evolutionary scenario of the mitochondrial genome in maize.

## Results

### Genome duplications

The analysis of maize and teosinte mitogenomes revealed the occurrence of duplications. Duplication length varied from 0.54 kbp to 120 kbp (Table 1). Duplicated fragments were an important part of the total genome length for the longest genomes, 23.4% for NA, 31.5% for CMS-C and 21.2% for *Zea mays *ssp. *parviglumis*, and more generally were the main cause of size differences among maize mitogenomes [24]. Six duplicated fragments were shared between maize [24] and *Zea mays ssp. parviglumis *mitogenomes : {NA, NB, CMS-C, CMS-S and *Zea mays *ssp. *parviglumis*} shared two duplications (11 and 17 kbp), {NA, NB, CMS-S and *Zea mays *ssp. *parviglumis*} a 0.7 kbp duplication, {NA, CMS-S, CMS-T and *Zea mays *ssp. *parviglumis*} a 5.3 kbp duplication, {NA, NB and *Zea mays *ssp. *parviglumis*} another 5.3 kbp duplication and {NA and *Zea mays *ssp. *parviglumis*} a 0.6 kbp duplication.

**Table 1:** Length and percentage of duplicated fragments up to 500 bp

Genome	Genome length (kbp)	Duplication **length (kbp)**^**a**^	% of dupl. fragments in genome	Minimal dupl. **length (kbp)**^**b**^	Maximal dupl. **length (kbp)**^**b**^	Median dupl. length (kbp)	Number of dupl. fragments	Genome length without duplication (kbp)
NA	701.046	163.899	23.4%	0.60	120.0	5.316	8	537.147
NB	569.630	49.407	8.7%	0.54	17.0	8.183	6	520.223
CMS-C	739.719	232.947	31.5%	5.70	105.0	31.009	6	506.772
CMS-S	557.162	45.023	8.1%	0.72	17.0	4.589	8	512.139
CMS-T	535.825	25.884	5.3%	2.60	12.8	5.270	4	509.941
parvi	680.603	143.928	21.2%	0.60	55.0	8.207	8	536.675
lux	539.368	18.561	3.4%	1.70	10.1	6.737	3	517.175
per	570.354	53.719	9.3%	6.30	13.6	11.809	5	520.807

^a^All duplicated copies less one

^b^Length of one copy

NA seemed to have a fragment duplicated in tandem, the two copies of its 120 kbp fragment were separated by only 9.3 kbp.

### Backbone and genome structures

#### Backbone DNA sequences

Total backbone DNA sequence (including genes) represented a concatenation of all common fragments between all mitogenomes when considering only one copy of each duplicated sequence. Overall, in maize, *Zea mays ssp. parviglumis, Zea perennis *and *Zea luxurians *mitogenomes, coding genes (including duplicated genes) represented 7.83 to 8.60% (median = 8.37%) of the total genome length whereas backbone DNA sequences represented 56.49 to 77.99% (median = 73.29%) of the total genome length (Table 2).

**Table 2:** Backbone, GSS (Genome Structure Sequence) and protein coding gene proportions on the mitogenomes

Genome	Mitogenome length (kbp)	% of protein coding gene in mitogenome*	% of GSS length in mitogenome*	% of backbone length in mitogenome*
NA	707.046	8.22	70.44	59.60
NB	569.630	8.44	73.60	73.36
CMS-C	739.719	8.23	73.61	56.49
CMS-S	557.162	8.37	74.21	75.02
CMS-T	535.825	8.36	71.58	77.99
parvi	680.603	7.83	72.74	61.41
lux	539.368	8.60	69.99	76.84
per	570.354	8.51	73.01	73.21

*including duplicates

**Figure 1:**
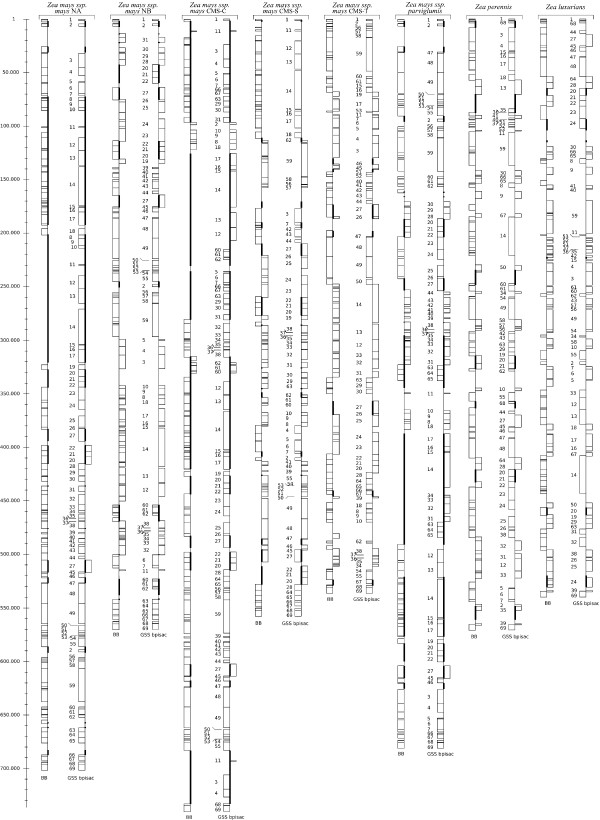
**Repartition of Backbone DNA sequences and Genome Structure Sequences (GSSs) Sequences on each mitogenome**. For each mitogenome, a pair of box sequences is represented : the backbone DNA sequence (BB) and the genome structure sequence before paralog identification and synteny anchor collapsing (GSS bpisac). Each box is either a Backbone DNA fragment for BB, or a synteny anchor for GSS bpisac. Boxes with the same number are homologous synteny anchors. The numbering of boxes was chosen according to *Zea mays *ssp. *mays *NA. Thus on BB and GSS bpisac, a box is drawn on the left side if it has the same orientation than its homolog in NA, on the right side otherwise. For each mitogenome, thick lines represent duplicated regions.

In all, there were 115 orthologous fragments over all mitogenomes (see Additional file 1). The smallest common fragment size was 94 bp and the largest was around 18,769 bp (median of 2,379 bp). Differences in size between orthologous fragments were due to indels (insertions and deletions). For each mitogenome, backbone sequence size was around 418 kbp, except for *Zea luxurians *with a size of 415 kbp. The multiple alignment length of the eight mitogenome backbones was 421,163 bp (counting gaps). Backbone repartition over *Zea *mitogenomes is given in Figure 1. We computed the gap sizes in the mitogenome sequences from the multiple alignment. Most of the gaps were 5 bp long as previously described by Allen and colleagues [24] and the insertions were small repetitions (data not shown). Compared to the other mitogenomes, *Zea luxurians *had more gaps longer than 5 bp. This mainly explains the backbone length difference between *Zea luxurians *and the other mitogenomes.

#### Genome structure sequence

The Genome Structure Sequence (GSS) is a block sequence characteristic of each mitogenome. It is built with block markers- which we hereafter call synteny anchors- that are common to all eight mitogenomes. Synteny anchors are composed of protein coding genes, tRNAs, rRNAs, ORFs, pseudogenes and non-coding sequences from the backbone DNA sequences (see Methods). Before paralog identification and synteny anchor collapsing ('bpisac'), GSSs contained 69 synteny anchors. They represented 69.99 to 74.21% of mitogenome lengths (median = 72.88%) (Table 2). Figure 1 provides a schematic view of GSS bpisac repartition over mitogenomes and shows that GSS bpisac uniformly covers all mitogenomes. It must be noted that in GSS, the numbers of synteny anchors correspond to one or more mitogenome markers: when they were systematically located together and in the same order in all eight mitogenomes, they were grouped into a single synteny anchor (see Additional file 2). Consequently there were 69 synteny anchors corresponding to 187 markers common to all mitogenomes. Synteny anchors contained from 1 (e.g. synteny anchor number 1) to 15 markers (e.g. synteny anchor number 59). As is generally the case in mitochondrial genomes, markers that were systematically grouped in our 8 mitogenomes were not composed of genes involved in the same metabolic pathway. Duplicated synteny anchors represented a large part of mitogenomes, particularly in NA, CMS-C and *Zea mays *ssp. *parviglumis*: 26.4% of the synteny anchors were duplicated in NA, 12.6% in NB, 36.8% in CMS-C, 12.6% in CMS-S, 2.3% in CMS-T, 20.7% in *Zea mays *ssp. *parviglumis*, and 9.2% in *Zea luxurians *and 10.34% in *Zea perennis*.

**Figure 2:**
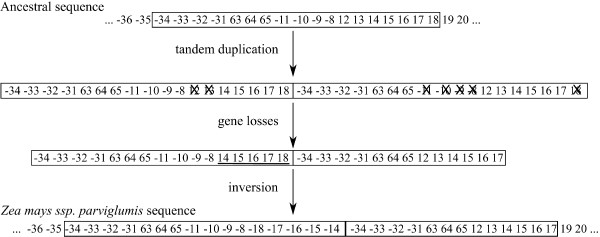
**Tandem duplication hypothesis**. A hypothetical scenario of evolution from an ancestral sequence to *Zea mays *ssp. *parviglumis *through tandem duplication, followed by deletions and inversions. Duplicated synteny anchors are written in bold face and the synteny anchors involved into the tandem duplication event are depicted in a frame box. Lost synteny anchors are stricked and inversion is underlined.

Using GSSs bpisac and assuming that tandem duplication was the underlying mechanism, we observed that most of the duplicated synteny anchors were indeed located in regions that could result from tandem duplication events. The fact that two regions did not share exactly the same synteny anchor content suggested deletion events of some duplicates after duplication. We called this mechanism Tandem Duplication with Partial Loss (TDPL). A hypothesis of TDPL in *Zea mays *ssp. *parviglumis *is shown in Figure 2.

**Figure 3:**
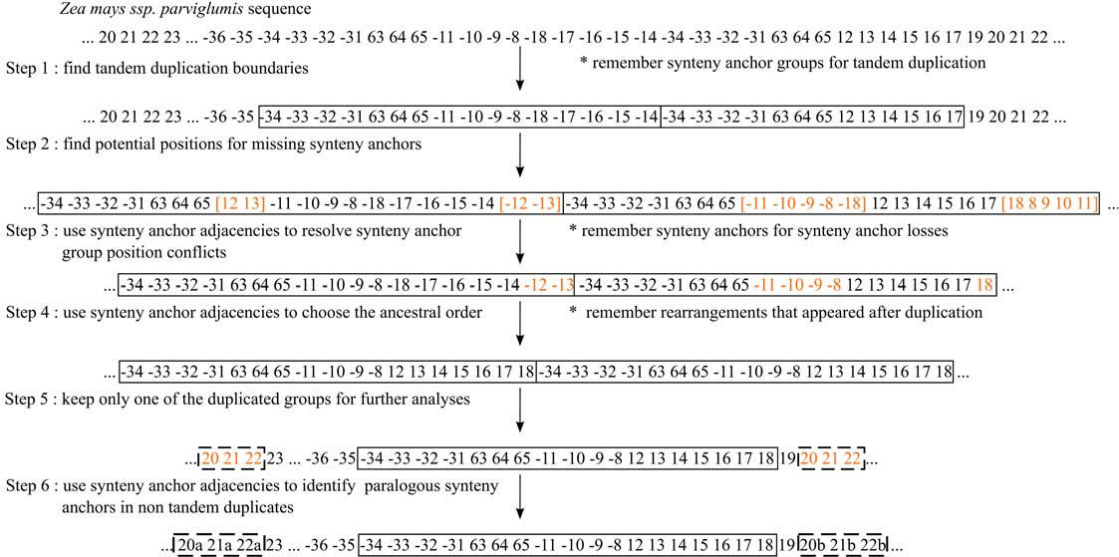
**Method to deal with duplicates**. Example of paralog identification and synteny anchor collapsing using *Zea mays *ssp. *parviglumis *GSS. Duplicated synteny anchors are in bold.

**Figure 4:**
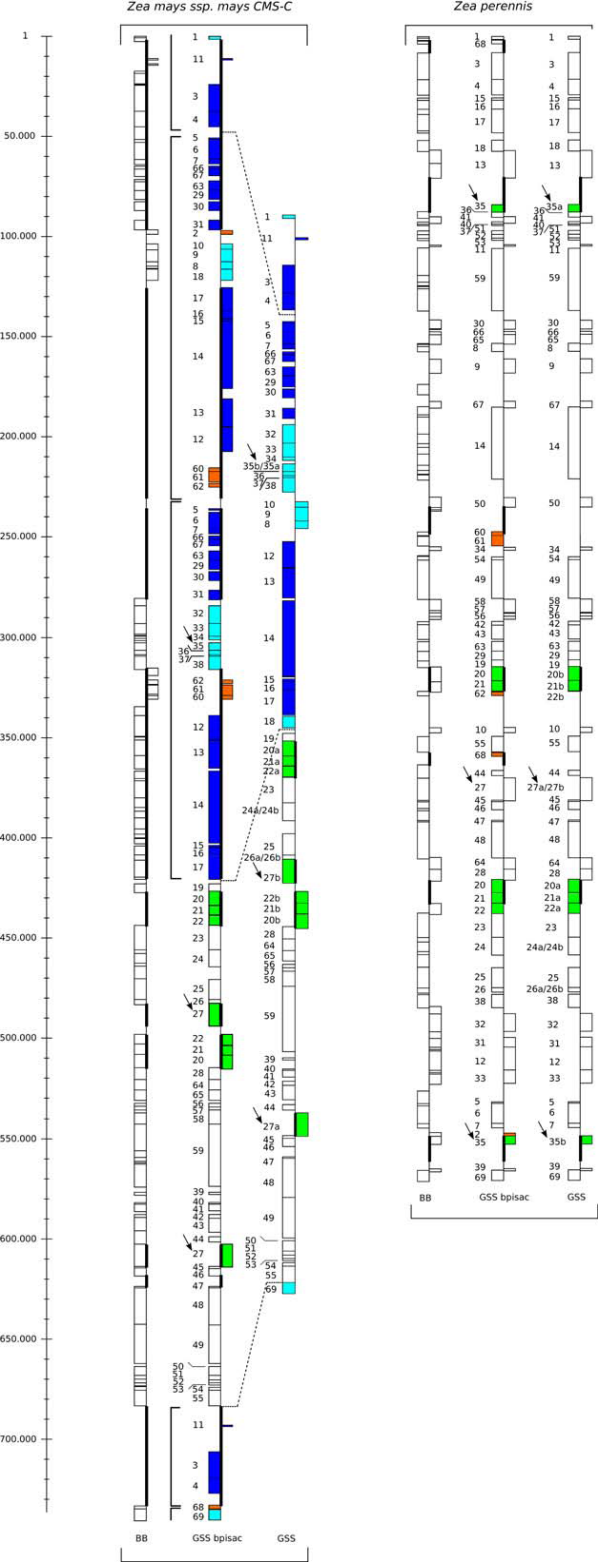
**Backbone DNA sequence, GSS bpisac and GSS**. Backbone DNA sequence (BB), GSS bpisac and GSS blocks repartition along CMS-C and *Zea perennis *mitogenomes. In CMS-C, dashed lines between GSS bpisac and GSS indicate the condensation of tandem duplicated synteny anchors (after the "collapsing" step). Vertical lines indicate each duplicated part. In CMS-C GSS, synteny anchors 35a and 35b are virtually added compared to GSS bpisac because synteny anchor 35 is duplicated in *Zea perennis *GSS bpisac and it is possible to distinguish them with their neighborhood. Conversely, 27 virtually duplicated (i.e. 27a and 27b are left side by side) in *Zea perennis *GSS is in two distinct copies in CMS-C GSS. For synteny anchor 40 duplicated in *Zea perennis *GSS bpisac, ortholog and paralog cannot be distinguished with their neighborhood. Consequently, synteny anchor 60, 61, 62 and 2 are deleted in GSS for all mitogenomes. Synteny anchors 27, 27a, 27b, 35, 35a and 35b are indicated by arrows. We applied the following color code: orange for deleted blocks, green for blocks for which paralogous from orthologous were distinguished, blue for tandemly duplicated blocks: dark blue when duplicates were conserved, light blue when one copy was lost.

Following the method described in Figure 3 and Methods (paralog identification and gene collapsing), GSS was obtained for each mitogenome, where duplicates were distinguished and/or collapsed. We identified 4 TDPLs specific to a mitogenome (one in NA, two in CMS-C and one in *Zea mays *ssp. *parviglumis*) where the two duplicates were still side by side, 2 TDPLs shared by some mitogenomes (one shared by all mitogenomes and the other by maize mitogenomes) where the two duplicates were physically separated and 4 tandem duplications specific to a mitogenome and where the copies were physically separated (CMS-S, CMS-T, *Zea luxurians *and *Zea perennis*). For these duplications, we hypothesized that the duplicates (originally in tandem) had been separated by rearrangement events after duplication. In the end, GSSs contained 72 blocks: the 69 original synteny anchors, minus 5 that were eliminated because orthologs and paralogs could not be distinguished, plus 8 additional blocks after paralog/ortholog identification. Transformation from GSS bpisac to GSS for CMS-C and *Zea perennis *is shown in Figure 4.

### Sequence phylogeny

**Figure 5:**
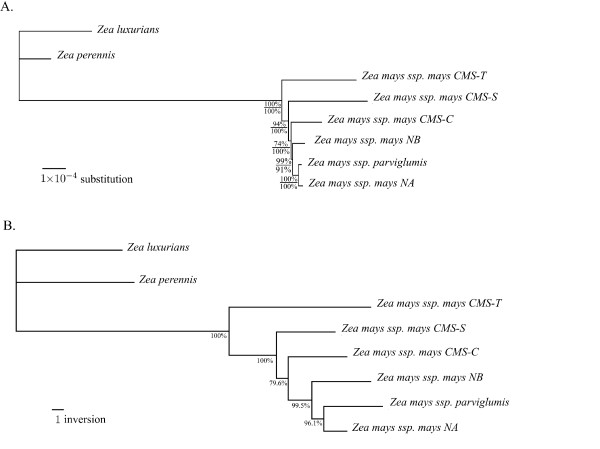
**Phylogenetic trees for maize and teosinte mitogenomes**. (A) Backbone DNA sequence phylogenetic tree. Phylogenetic tree was constructed using BIONJ and TREE-PUZZLE. The tree was rooted using *Zea perennis *and *Zea luxurians*. Branch lengths are proportional to substitution rates. Bootstrap values (upper values for distance and lower values for likelihood) are reported. (B) Structure sequence phylogenetic tree. Phylogenetic tree was constructed using BIONJ from the number of inversion distances obtained with GRIMM. The tree was rooted using *Zea perennis *and *Zea luxurians*. Branch lengths are proportional to the numbers of inversions. Jackknife values are reported.

Bootstrap values (upper values in Figure 5.A.) indicated that the topology of the tree was relatively robust (from 94-99%) with some uncertainty regarding the separation between CMS-C and the remaining three *Zea mays *mitogenomes (74%). The Maximum Likelihood (ML) phylogenetic tree had the same topology as the NJ phylogenetic tree and bootstrap values (lower values in Figure 5.B.) were higher for all nodes. Molecular clock with ML was rejected (p < 0.0001).

We also constructed a phylogenetic tree with concatenated protein coding gene sequences which exhibited the same topology as the one from the backbone sequence but with shorter branch lengths (data not shown).

### Rearrangement phylogeny

Phylogenetic analysis was based on rearrangement using GSSs. The phylogenetic tree was congruent with the one from backbone DNA sequence. Jackknife values were 96.1%, 99.5%, 79.6%, 100% and 100% for the five most terminal nodes (Figure 5.B). Tests were performed with different percentages of synteny anchors kept in the jackknife computation (see Additional file 3). When 30 to 90% of the synteny anchors were kept, the main tree was congruent with the sequence tree.

We built a phylogenetic tree with a data set excluding blocks containing duplicated synteny anchors. It is noteworthy that the resulting tree was not congruent with the sequence tree. This highlights the importance of taking into account duplication events in the analysis. Moreover, when we deleted all copies of each duplicated synteny anchors, the data set went down from 72 to 28 synteny anchors.

### Mitogenome rearrangement evolution

**Figure 6:**
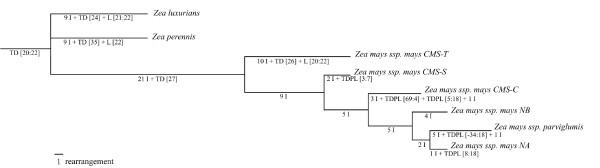
**Maize and teosinte mitogenomes evolutionary tree**. Parsimonious phylogenetic tree (built with MGR) using GSSs. Branch length is proportional to the number of rearrangement events (inversion, duplications, deletions). An Ancestral sequence is given for each node (A1 to A5). Rearrangement events are given on each branch: I = inversion, TD = tandem duplication, L = synteny anchor loss, TDPL = tandem duplication with partial loss. Numbers between vertical lines correspond to blocks affected by rearrangements.

A parsimonious tree was constructed using MGR (Multiple Genome Rearrangements) with GSSs. This method has the advantage of providing a potential ancestral sequence at each node (A1 to A5) (Figure 6).

**Figure 7:**
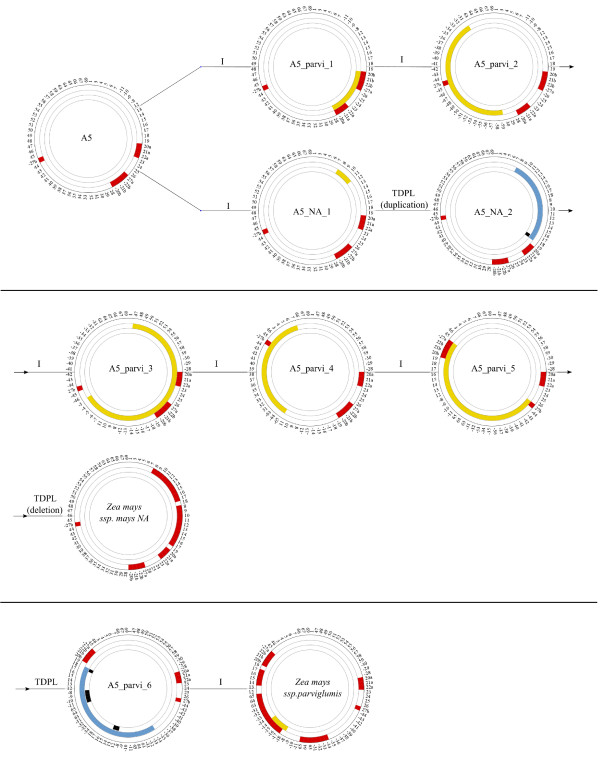
**Predicted evolution from A5 to NA and *Zea mays *ssp**. ***Parviglumis***. Example of an evolutionary scenario from A5 to *Zea mays *ssp. *mays *NA and *Zea mays *ssp. *parviglumis*. A5 (the ancestral sequence computed by MGR) and intermediate mitogenomes after each rearrangement event are shown. Evolution of A5 mitogenome through inversions, tandem duplications (respectively yellow and blue in the middle circle) and synteny anchor loss (black in the internal circle) leads to NA (with 2 rearrangements) and *Zea mays *ssp. *parviglumis *(with 7 rearrangements). Duplicated synteny anchors are in red (outside circle). On each branch, the rearrangement event is indicated (I = Inversion, TDPL = Tandem Duplication with Partial Loss). For NA, the TDPL is indicated in two steps: a duplicated event followed by a deletion event.

It was possible to reintroduce duplication events in the MGR tree. Indeed, duplication of synteny anchors {8 9 10 11 12 13 14 15 16 17 18} can be put on the NA branch, duplication of synteny anchors {5 6 7 66 67 63 29 30 31 32 33 34 35 36 37 38 -10 -9 -8 12 13 14 15 16 17 18}and {69 1 -11 3 4} on the CMS-C branch and duplication of synteny anchors {-34 -33 -32 -31 63 64 65 -11 -10 -9 -8 12 13 14 15 16 17 18} on the *Zea mays *ssp. *parviglumis *branch. These duplication events were followed by synteny anchor loss and inversions (as described in Figures 2 and 3). It was then possible to obtain a parsimonious evolutionary history of all eight mitogenomes. Likely events were positioned on each branch of the tree where (I) denotes an inversion, (TD) a tandem duplication, (TDPL) a tandem duplication with partial loss and (L) a loss. In Figure 7, an example of an evolutionary scenario is given from A5 to *Zea mays *ssp. *parviglumis *and NA.

It must be noted that some rearrangement events need not occur in an absolute order except for overlapping inversions, TDPLs and the last inversion in CMS-C and *Zea mays *ssp. *parviglumis*. It appears that overlapping inversions must be chronologically oriented in the evolution history: for example, from A5 to *Zea mays *ssp. *parviglumis*, inversion I{-31:-59} has to occur before inversion I{47:-3}. However, non-overlapping inversions can be permuted: for example, I{-20b:-20a} can occur either before or after I{-31:-59}. Two duplications have an ancestral position: TD{20:22} is ancestral to maize and teosinte mitogenomes and TD{27} is specific to maize mitogenomes (Figure 6). Over time, the duplicates were separated.

It is important to note that scenarios for all mitogenomes, computed by MGR, were consistent with rearrangement sites (i.e. breakpoint regions) observed at the sequence level by Allen and colleagues [24]. Indeed, many rearrangements predicted by MGR occurred between the second copies of synteny anchors 20 and 21 (*trn*N and orf99 in the region 140 kbp of NB); we also found rearrangement points near synteny anchors 4, 7, 9 and 18 (respectively *cob*, *nad*2 exon 1, *rbc*L and *cox*1 genes) whereas we did not find any rearrangements between synteny anchors 27 (first copy) and 21 (second copy) (*nad*1 exon 1 and *rps*3 exon 1 in the region 65 to 140 kbp of NB).

## Discussion

We analyzed the evolution of mitochondrial genome structure within a plant species by concomitantly building a phylogenetic tree based on sequence polymorphism and a phylogenetic tree based on structural rearrangements among genomes. Both trees were congruent, suggesting that both sources of polymorphism are correlated, i.e. the more divergent a genome is, the more rearranged it is. Therefore it was possible to reconstruct an evolutionary scenario, suggest ancestral genome structures along the different nodes of the tree, and pinpoint tandem duplication as a possible mechanism in the important gene shuffling of plant mitochondrial genomes.

### Methodology to deal with duplicates

From a methodological point of view, dealing with duplicates together with rearrangement events is a challenge. If one was able to distinguish between paralogous and orthologous synteny anchors, the problem would be reduced to the study of rearrangements with exactly one copy of each synteny anchor in each genome. Unfortunately, finding paralogous synteny anchors is usually a very difficult task (this is especially the case with the data analyzed here since mitochondrial synteny anchor duplicates are identical in most cases). Even if one was able to distinguish them, it remains that some duplicates are specific to a given genome or to a subset of genomes. Different methods have been proposed to deal with such datasets. In the exemplar model [20], only one copy of each duplicate is kept. In the maximum matching model [25], one keeps as many copies as the minimum number of copies of one duplicate found in a genome. The choice of which copy to keep is made according to an optimization function. For genome rearrangement purposes, this function consists in choosing the copies that minimize the evolutionary distance between two genomes. But such methods can be applied only if the number of duplicates remains small, otherwise the number of reduced genomes is too large. This is the case with our data. The exemplar genome approach would have led us to explore more than 16 million datasets from our eight mitogenomes. In the special case of tandem duplications, a method was previously described with random loss (TDRL) [26]. Unfortunately, in that model exactly one of each duplicate is immediately lost just after the duplication event, and the method proposed cannot be adapted because the underlying algorithms require that each marker synteny anchor be present only once in each genome.

Therefore we proposed a framework to analyze the rearrangement history of a set of genomes containing duplicates. In this framework we assumed that most of the duplicates came from tandem duplication events and that rearrangements occurring within a duplicated segment were independent from the other rearrangement events. Although this is not necessarily true in the general case, we found evidence of tandem duplication in parts of the genome. These hypotheses provided a means to deal with duplications and to allow us to propose both a scenario of rearrangements and a history of duplication events. We thus elaborated a four step method to account for duplicates. In short, we concealed duplicates to compute rearrangement scenarios and then we reintroduced them. The method was the following : i) identify TDPLs and collapse them in order to keep one copy of each synteny anchor, ii) distinguish between paralogs and orthologs for remaining duplicated synteny anchors, iii) apply the usual rearrangement algorithms (since no duplicate remains), iv) expand the previously collapsed TDPLs in step i) to recreate the TDPL event. The main difficulty of the first step is to correctly determine the boundary of the duplicated segment. We saw that using the information of the synteny anchor neighborhood shared by the genomes could help determine these boundaries (see Methods and Additional files 4). The second step proved to be more difficult since we had to deal with the problem of ortholog versus paralog identification. We supposed that the number of duplicated blocks involved in a TDPL but far apart from each other was rather limited and that the methods described above could thus be used. In this last case, though, the neighborhood could help distinguish between both duplicates (such as block 27 in the dataset). When the duplicates were not in tandem, we added the duplicated block in tandem with its counterpart in genomes in which it was missing because the block content had to be the same for the third step of the method. This did not change the distances among genomes nor did it modify scenarios. Indeed, adding the duplicated block next to its counterpart created an adjacency that was implicitly conserved when computing parsimonious scenarios. The last step consisted of replacing the collapsed TDPLs by their original block sequences. The duplication events were placed on the tree depending on whether TDPL was shared by several genomes or not. If the TDPL was specific to one genome, the duplication event necessarily occurred after the last speciation event. If a TDPL was shared by two or more genomes, the most parsimonious hypothesis was that the duplication event occurred just before the speciation event.

### Phylogenetic relationships among Zea mitochondrial genomes

The phylogenetic relationships among maize mitogenomes concord with a former study by Allen and colleagues [24] where NA and NB were described as being the most-closely related mitogenomes, followed by CMS-C, CMS-S and CMS-T. On the basis of their nucleotide divergence, CMS-S and CMS-T were suggested to be the oldest cytoplasms. The introduction of two additional mitogenomes from the outgroup species of teosintes *Zea luxurians *and *Zea perennis *also suggested the ancestral position of CMS-S and T. Former studies on mitochondrial and chloroplastic diversity in *Zea *pointed out the fact that CMS-S was an old cytoplasm and most likely the result of introgression from teosinte *Zea mays *ssp. *mexicana*. But the phylogenetic location of CMS-T, due to a strong nucleotide divergence and a concomitant rearranged genome, is puzzling since CMS-T shares the same co-inherited chloroplastic genome with CMS-C and NB [27,28]. Consequently, the high divergence of CMS-T might not have occurred in a molecular clock tempo (as suggested by the rejection of the molecular clock hypothesis in the phylogenetic analysis). Chloroplastic sequence data could shed light on the relative ages of the cytoplasms studied. It is interesting to note that the same phenomenon was observed when considering the chloroplastic nucleotide diversity among several cytoplasms of wild beet: cytoplasm *Nv *and CMS *Owen *are closely related when considering chloroplastic nucleotide divergence [29] while mitochondrial genomes are highly rearranged and exhibit about 8% of specific sequences [30].

The phylogenetic location of *Zea mays *ssp. *parviglumis *included in the *Zea mays *clade concords with the scenario of a recent maize domestication from this teosinte subspecies [31]. Moreover, it highly suggests that the cytoplasms we studied differentiated before domestication.

### Tandem duplication with partial loss as a plausible mechanism

Tandem duplication is a mechanism that has been demonstrated or at least suggested in mitochondrial genomes of several animal species, even though the underlying molecular mechanism is not always understood [32,33]. Tandem duplications have been mainly observed in Chordata, particularly in Vertebrates such as Lizards [33], Salamanders [9], Amphibians [34] or Gulper Eels [8]. Cases of tandem duplication are not restricted to Chordata, they have also been reported in Echinodermata [10], Insecta [35] and Lophotrochozoa (e.g. Mollusca) [36-38]. It must be noted that different types of tandem duplication have been observed in all these species: duplications of the whole genome, tandem duplications of genome parts, tandem duplications of non-coding regions or tandem duplications of one gene. In most cases, only one functional copy of the duplicates remains after duplication.

Mitochondrial genomes of maize and teosintes (*Zea mays *ssp. *parviglumis*, *Zea luxurians *and *Zea perennis*) could undergo the same mechanism of tandem duplication with loss as animal mitochondrial genomes. A possible mechanism could rely on the integration in the master chromosome of minicircles generated by homologous recombination between direct repeats from the original master circle, resulting in a duplication event [39]. But this would imply a preferential adjacent integration (see discussion by Fujita and colleagues [33] for animals). The low substitution rate in the maize mitogenome may explain why, in maize mitogenomes, one or more copies of duplicated synteny anchors remain, as opposed to animal mitogenomes where all gene copies but one are lost. More generally, a causal link has been suggested between mutation rate and genome compactness that could explain the large size and gene duplicate occurrence of plant mitochondrial genomes when compared with their animal counterparts [2]. The fact that the same mechanism could be involved in mitochondrial genomes of plants and animals falls in line with the monophyletic origin of animal and plant mitochondrial genomes [1]. For example, red algae [40], that form an independent lineage that radiated contemporarily with the other evolved eukaryotic lineages, demonstrates characteristics of both plant (gene with introns, ribosomal proteins) and animal mitochondria (modified genetic code, short mitochondrial sequence). Similar observations have been made for *Acanthamoeba castellanii *[41] or *Trichoplax adhaerens *[42].

Looking at the literature from the past decades, emphasis has been put on differences between animal and plant mitogenomes in their evolutionary dynamics and at the structure level [11,14]. While a compact circular genome is found in the majority of animal lineages, the plant mitogenome was described as a dynamic equilibrium of isoforms of a master circular chromosome and sub-molecules due to the occurrence of repeated sequences favoring intragenomic recombination. In this context, it is particularly interesting to notice that the evolutionary scenario based on rearrangement among master circles is congruent with the analysis based on sequence divergence among them. Therefore, it appears that master circles might reflect more than a virtual synthetic representation.

## Conclusions

Despite important structural shuffling among genomes, even at the species level, we were able to build a phylogenetic tree using rearrangement events between plant mitochondrial genomes that was congruent with a sequence-based tree. To our knowledge this is the first evolutionary scenario of a plant mitogenome proposed solely on the basis of rearrangement events in complete DNA sequences. We showed that, under the hypothesis of structure evolution through inversions and tandem duplications with loss, an evolutionary path could be drawn for each genome. While such evolutionary events have been identified in animal mitogenomes, the hypothesis of a similar mechanism has never been discussed for plant mitogenomes. Further work will consist of developing new tools in order to automatically look for signatures of tandem duplication events in other plant mitogenomes and evaluate the occurrence of this process on a larger scale.

## Methods

### Data

#### Mitochondrial genomes used

The eight studied mitogenomes from *Zea *were downloaded from GenBank. Among the 5 recently sequenced mitogenomes from *Zea mays subsp. mays*, two of them are fertile cytotypes *NA *[GenBank:DQ490953] and *NB *[GenBank:AY506529], and three of them are cytoplasmic-male-sterile (CMS) cytotypes: *CMS-C *[GenBank:DQ645536], *CMS-S *[GenBank:DQ490951] and *CMS-T *[GenBank:DQ490953] [24]. We enriched the dataset with the mitogenomes of three teosinte species, *Zea mays subsp. parviglumis *[GenBank:DQ645539], *Zea luxurians *[GenBank:DQ645537] and *Zea perennis *[GenBank:DQ645538] (Allen *et al.*, unpublished results). The two last mitogenomes served as outgroups for phylogenetic analysis. Table 1 summarizes the genomes used.

We noted that all mitogenomes are in the master circle conformation and all our analyses were based on this conformation.

#### Synteny blocks

Synteny blocks, representing conserved sequence blocks between all mitogenomes, were computed using Mauve [43], a tool performing multiple genome alignments between sequences that can be rearranged. Mauve uses a set of genome DNA sequences as input. It locally computes co-linear blocks from anchors that are short unique similar DNA fragments. The anchors are then extended in order to produce longer common segments. Finally, the segments are clustered to locally produce co-linear blocks under the constraint that, for a given genome, segments have to be on the same strand. As the Mauve algorithm keeps short unique similar DNA fragments, duplicated DNA sequences are not taken into account. Mauve provides a backbone file containing synteny blocks and an alignment file containing the alignments of each synteny block.

Mauve parameters used are match weight seed = 9, minimum island = 15, maximum backbone gap size = 15, minimum backbone size = 50. Match weight seed parameter is essential in the multiple alignment and depends on the number of genomes to align and their lengths. Default weight seed is 11 for genomes of 1 MB length and increases with the genome size. As mitogenomes used in this study have a size comprised between 535 and 740 Kb, we set the weight seed at 9 (lower values were tested but a weight seed of 9 provided the best results). Minimum island is the minimum size for a fragment that is not common to all genomes. Maximum backbone gap size is the maximum size authorized for a gap in sequences common to all mitogenomes. If one mitogenome had a gap longer than to 15 bp in a sequence block, this block was split into two blocks at the gap. Minimum backbone size is the minimum size for a sequence block.

#### Backbone DNA sequence

In order to compare mitogenomes at the sequence level, for each genome we used the backbone and the alignment sequences provided by Mauve to build a sequence made of the concatenation of the synteny blocks, called *backbone DNA sequence*. As duplicated sequences are not taken into account in Mauve, we masked one copy of each duplicate (size >500 bp) for each mitogenome. A reference genome was chosen (here NA) in order to build the backbone DNA sequences. For each genome, the synteny blocks were concatenated, following the order of the synteny blocks on the reference genome. As we kept all common sequences between the eight genomes, the choice of one reference genome instead of another does not change the results. As the method used for computing synteny blocks allows insertions, deletions and substitutions, the length of a synteny block may vary depending on the genome and therefore the length of the backbone sequence may be different for each genome. The number of synteny blocks and the length of the backbone sequence for the eight genomes were summarized in Additional file 1. The repartition of synteny blocks for the mitogenomes was provided in Figure 1.

#### Genome structure sequence

In order to study genomic rearrangements we had to build a genome structure sequence (i.e. genome marker order) out of the genome DNA sequence. Such a genome structure sequence is an abstraction of the genome seen as a sequence of blocks that can be rearranged. The main difference when compared with the backbone sequence is that the DNA sequence within each block is no longer considered.

To build the genome structure sequence of each mitogenome, we applied the following strategy: i) first, we extracted annotated protein coding genes, tRNAs, rRNAs, ORFs (Open Reading Frame) and pseudogenes from the corresponding GenBank file, and then, ii) non-coding sequences from the backbones.

For coding sequences extracted from all eight mitogenomes we built a database. For each sequence, we used the YASS (Yet Another Similarity Searcher) software [44] against this database (excluding the sequence of interest). We conserved all reciprocal best hits in order to identify orthologous markers. As E-value depends on the sequence lengths compared, different E-values were used when sequences were shorter or longer than 100 bp. For the case of protein coding genes, rRNAs, ORFs and pseudogenes (with a length higher than 100 bp), we considered only RBHs with an E-value lower than 1*e*^-170 ^and with an alignment length difference of less than 8%. For the case of tRNAs and some protein coding gene exons (with a length shorter than 100 bp) we chose an E-value of 1*e*^-26 ^and an alignment length difference of less than 8%. When it was impossible to distinguish between two reciprocal best hits (same E-value and same sequence length), the copies were considered as homologous. If a marker was missing in a genome, we launched a search using YASS in order to check if it was a misannotation. If the marker was not found, the homologs (orthologs and paralogs) in other mitogenomes were excluded from the study.

For non-coding sequences, we used fragments from the backbone sequences that were larger than 100 bp. We did not consider those included in a coding region (because they would have been counted twice in the dataset). Using the YASS software, we only kept duplicates with an alignment length difference of less than 8%.

We thus obtained a set of 187 markers common to all genomes. If markers were found in the same order in all mitogenomes, we grouped them into marker groups, their boundaries corresponding to the flanking markers. Overall, the extraction procedure resulted in a total of 69 markers along mitogenomes that we call hereafter *synteny anchors*.

We obtained synteny anchor structure sequences by assigning a number to each synteny anchor. Using NA as the reference genome, each synteny anchor was assigned a number in ascending order from left to right. The numbering of the other genomes was based on NA (using another reference mitogenome does not change the results). A plus or minus was assigned to each synteny anchor depending on the strand where the synteny anchor occured in the NA genome. These structure sequences, where synteny anchor orthologs and paralogs had the same number, were called GSS bpisac (Genome Structure Sequence before paralog identification and synteny anchor collapsing). Additional file 2 provides the composition and numbering of synteny anchors used to build the GSS for each genome, Figure 1 depicts GSS bpisac blocks repartition along the eight genomes.

In order to test our hypothesis of tandem duplication in maize and teosinte mitogenomes, we needed to take into account duplicated synteny anchors. As paralogous synteny anchors have identical nucleotide sequences, we used the neighborhood graph (see below and Additional file 4) to distinguish them. Two different duplication types (of one or more synteny anchor groups) could be observed : unique to a mitogenome or shared by some or all mitogenomes.

If a duplication was specific to one genome and seemed to be tandem duplicated, we considered it as being a recent event. In order to integrate the duplicated synteny anchors in the dataset, we first looked for the bounds of the duplicated part, then we reintroduced all deleted synteny anchors yielding two juxtaposed identical parts, and finally collapsed the synteny anchors involved in the two parts by re-numbering them to obtain the part before tandem duplication.

If a duplication was shared between genomes (or specific to one genome and not tandem duplicated), we considered that there was a tandem duplication at the ancestral level. When synteny anchor copies were distant along the mitogenomes, we decided to distinguish the copies using their synteny anchor adjacencies in the eight genomes.

Through the neighborhood graph and the resulting hierarchical clustering (see Additional file 4) made on GSS before paralog identification and synteny anchor collapsing (bpisac), we determined the bounds of each duplicated part (duplicates are on a thick line on GSSs bpisac in Figure 1 and Figure 4). For example, for CMS-C, it was difficult to choose if synteny anchors {32 33 34 35 36 37 38} had to be clustered with {31} or with {60}. Thanks to the hierarchical clustering, {32 33 34 35 36 37 38} was put with {31} because {32 33 34 35 36 37 38} were clustered with {31}. After all obvious tandem duplications were collapsed, some duplications remained. Some of them were specific to a given mitogenome, while the others were shared by several mitogenomes. In the case of {20 21 22}, for which at least one copy was found in all mitogenomes, we made the hypothesis of an ancestral duplication of this group followed by loss of one copy of {21 22} in *Zea luxurians*, one copy of {22} in *Zea perennis *and all copies in CMS-T. Other mitogenomes had kept all copies. We renumbered one of the duplicates, depending on the neighborhood. For example, {20 21 22} was associated with {23 24 25 26}, that is why the first occurrence of {20 21 22} next to {23 24 25 26} was renumbered {20a 21a 22a} and the other occurrence was renumbered {20b 21b 22b}. We did the same for the group {27}, one copy (next to {44}) was renamed {27a} and the other was renamed {27b}. If a synteny anchor was duplicated (not in tandem) in only one mitogenome, we also distinguished the two occurrences. Under the postulate of a tandem duplication event specific to this genome, we added the new number in tandem with the first occurrence in the other mitogenomes. This ensured that GRIMM kept synteny anchors together when computing evolving scenario between all other mitogenomes. It was the case for {24} in *Zea luxurians*, {26} and {67} in CMS-T, and {35} *Zea perennis *where paralogs were respectively renumbered {24b}, {26b},{67b} and {35b}. All duplicated synteny anchors were then distinguished except for {2} duplicated in NA and NB, {60, 61, 62} duplicated in NB, and {68} duplicated in *Zea perennis*. All copies of these five synteny anchors were thus deleted from the dataset.

It was thus possible to distinguish between paralogs and orthologs for 8 out of 13 duplicated synteny anchors (see Figure 3).

Then we were able to apply known rearrangement methods on this structure called GSS. The GSS was composed of 72 synteny anchors. Figure 4 provides a comparison of GSS bpisac and GSS for CMS-C and *Zea perennis *mitogenome.

#### Neighborhood graph and synteny anchor clusters

Neighborhood relationships between synteny anchors were modeled in a graph. Two synteny anchors were considered to be in the same neighborhood if they were separated by at most one synteny anchor. A weight function was defined between two synteny anchors as the number of times both synteny anchors were neighbor. For a given weight *w*, a cluster of synteny anchors was defined as a set of synteny anchors such that: i) for any synteny anchor *s *in the set there exists another synteny anchor *s' *such that *s *and *s' *are neighbor and the value of the weight function between them is greater than *w*, ii) for any synteny anchor *s *in the set and for any synteny anchor *s' *outside the set, *s *and *s' *are not neighbors or they are neighbors but the value of the weight function between them is lower than *w*. That is two synteny anchors were in the same cluster if they were separated by at most one synteny anchor at least *w *times. We used this definition of synteny anchor cluster because usual gene clusters such as common intervals [45] or gene teams [46] cannot be applied to our data: the definition is too restrictive and/or does not support duplicated genes.

### Sequence analysis

#### Method for counting duplicated segments

Mitogenome statistics were performed with an in-house script using YASS in order to detect large duplicated segments (longer than 500 bp). YASS aligns pairwise sequences and finds conserved segments. As we were looking for highly conserved segments, we used a score of +1 for matches and a score of -3 for substitutions. Segments up to 500 bp (as in [24]) and with an E-value lower than 1e^-300 ^were considered as paralogous.

#### Substitution rate

Sequence substitution rates were computed from the backbone DNA sequences and protein coding gene sequences for each mitogenome pairs. Protein coding gene sequence is the concatenation of one copy (since the copies are identical) of each protein coding gene, common to all mitogenomes. Substitution rate (for 10 kb) between two genomes was calculated as follows :

Ratio of substitution rates between backbone DNA sequences and protein coding genes was also calculated (Table 3).

**Table 3:** Ratio of pairwise genome substitution rate between backbone and protein coding sequences per 10 kb

	NB	CMS-C	CMS-S	CMS-T	parvi	lux	per
NA	4.002	1.296	1.428	0.994	1.770	1.453	1.299
NB	-	1.990	1.729	1.176	2.694	1.553	1.393
CMS-C	-	-	1.653	1.115	1.122	1.544	1.375
CMS-S	-	-	-	1.360	1.355	1.743	1.548
CMS-T	-	-	-	-	0.948	1.220	1.149
parvi	-	-	-	-	-	1.411	1.270
lux	-	-	-	-	-	-	1.055

### Structure sequence analysis

A simple way to measure a rearrangement distance between genomes is to count the number of breakpoints [47-49]. A breakpoint is a disruption of the genome sequence order, i.e. when adjacency between two genes in one genome disappears in another one. A breakpoint matrix distance among genomes provides a way to reconstruct a phylogenetic tree using distance methods [50]. But such a basic tool does not provide any information about the history of rearrangements.

To further pursue the analysis of genomic rearrangements, one might compute the rearrangement distance as the minimal number of rearrangement operations needed to transform a genome into another [51]. This distance can also be used to build a phylogenetic tree : the more similar two genomes are, the smaller the rearrangement distance between them. The computation of such a distance also provides the scenario of operations that rearranged a genome into another. This allows one to build parsimonious phylogenies and propose ancestral nodes [18]. We used the GRIMM software (Genome Rearrangements In Man and Mouse -this software is not specific to Human and mouse genomes) [52] to compute inversion distances and scenarios. This software computes parsimonious inversion scenarios given a set of genomes as sequences of numbers without duplicates.

### Phylogenetic analysis

#### At the DNA sequence level

Neighbor-Joining analyses were realized on the backbone DNA sequences using BIONJ [53]. Parameters used are bootstrap 1000× and Kimura-2 parameters distance for correction. Maximum likelihood and molecular clock were tested with TREE-PUZZLE [54] using the nucleotide model of Hasegawa-Kishino-Yano (HKY85) [55].

#### At the structure sequence level

Rearrangement analyses were performed using GRIMM onto the GSSs. We obtained a distance matrix and then used BIONJ on this matrix to obtain a phylogenetic tree. Unfortunately, no bootstrap method is available for rearrangement studies. In order to test the robustness of the reconstructed trees, we adapted a Jackknife test [56,57] on the GSSs as follows: we randomly kept ninety percent of the GSS blocks (65 blocks out of 72); on this subset we computed a GRIMM matrix and we built a phylogeny using BIONJ; 1000 tests were applied. We thus obtained 1000 trees. We reported the frequency of the nodes found in the original tree according to this set of trees. We performed tests for several percentages of kept GSS blocks (10%, 20%,...100%) using the same method (see Additional file 3). The MGR (Multiple Genome Rearrangements) software [18] answers the problem of computing a parsimonious phylogeny given a set of genomes represented as sequences of numbers without duplicates. Unfortunately this problem has been shown to be computationally hard (NP-hard). It follows that MGR provides an approximate solution which is often near optimal [18].

## Authors' contributions

AD, JSV and PT designed the study. AD ran all the analyses and prepared all figures and tables. AD, JSV and PT interpreted the results and wrote the manuscript. All authors read and approved the final manuscript.

## Supplementary Material

Additional file 1 **Backbone DNA fragments**. Each orthologous fragment between mitogenomes is represented by an arrow. Fragment with the smallest size is underlined in blue and fragment with the longest size in red. Click here for file

Additional file 2 **Synteny anchor numbers and compositions**. Synteny anchors contained in GSSs. A synteny anchor often contains more than one genome marker (gene, tRNA, rRNA, ORF, pseudogene or non-coding sequence from backbone DNA sequence). Click here for file

Additional file 3 **Jackknife tests**. Node values for percentage of conserved GSS blocks. For each percentage of conserved synteny anchors, 1000 GRIMM matrices were computed and 1000 trees were drawn from these matrices. Each node value obtained for the consensus of these 1000 trees was reported in the graph. For example, for 90% of conserved GSS synteny anchors, Jackknife value for the terminal node (separation between NB and the remaining two *Zea mays *mitogenomes) 96.1%. Click here for file

Additional file 4 **Hierarchical clustering**. Hierarchical clustering obtained with the neighborhood graph using GSSs. Two synteny anchors closer to one another than the others were assigned to the same cluster. Click here for file
