# Supplementary figures and images for: A scenario of mitochondrial genome evolution in maize based on rearrangement events
Source: BMC Genomics. 2010 Apr 9;11:233. doi: 10.1186/1471-2164-11-233 (PMC2859866; doi:10.1186/1471-2164-11-233)

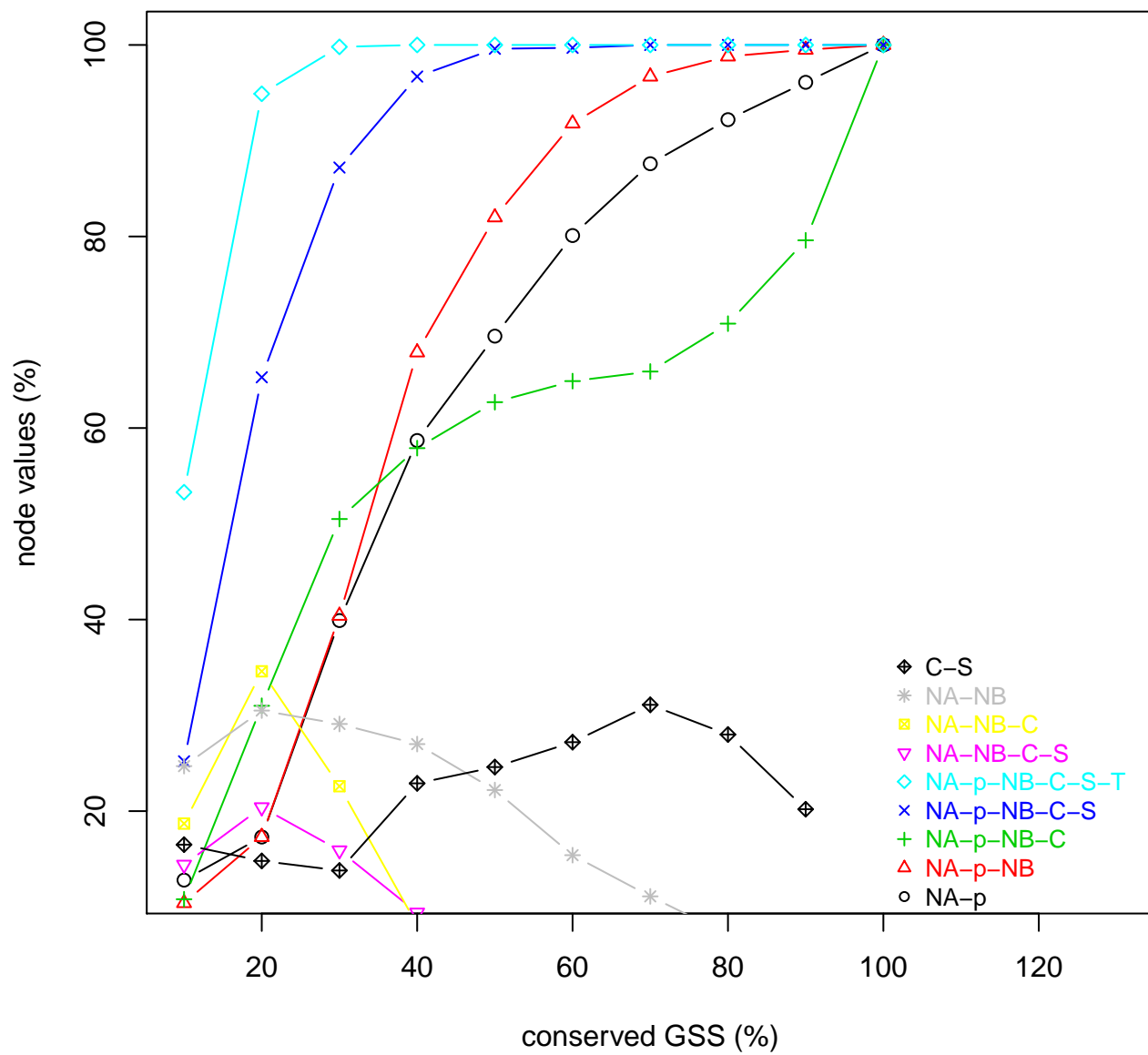

Supplement: Additional file 3 — Jackknife tests. Node values for percentage of conserved GSS blocks. For each percentage of conserved synteny anchors, 1000 GRIMM matrices were computed and 1000 trees were drawn from these matrices. Each node value obtained for the consensus of these 1000 trees was reported in the graph. For example, for 90% of conserved GSS synteny anchors, Jackknife value for the terminal node (separation between NB and the remaining two Zea mays mitogenomes) 96.1%. [file 1471-2164-11-233-S3.PDF]
